# Trading amino acids at the aphid–*Buchnera* symbiotic interface

**DOI:** 10.1073/pnas.1906223116

**Published:** 2019-07-23

**Authors:** Honglin Feng, Noel Edwards, Catriona M. H. Anderson, Mike Althaus, Rebecca P. Duncan, Yu-Ching Hsu, Charles W. Luetje, Daniel R. G. Price, Alex C. C. Wilson, David T. Thwaites

**Affiliations:** ^a^Department of Biology, University of Miami, Coral Gables, FL 33146;; ^b^Institute for Cell & Molecular Biosciences, Faculty of Medical Sciences, Newcastle University, NE2 4HH, Newcastle upon Tyne, United Kingdom;; ^c^School of Natural & Environmental Sciences, Newcastle University, NE1 7RU, Newcastle upon Tyne, United Kingdom;; ^d^Department of Molecular and Cellular Pharmacology, Miller School of Medicine, University of Miami, Miami, FL 33136

**Keywords:** symbiosis, amino acid transport, metabolic integration

## Abstract

Plant sap-feeding insects are widespread, having evolved to occupy diverse environmental niches despite exclusive feeding on an impoverished diet lacking in essential amino acids and vitamins. Success depends exquisitely on their symbiotic relationships with microbial symbionts housed within specialized eukaryotic bacteriocyte cells. Each bacteriocyte is packed with symbionts that are individually surrounded by a host-derived symbiosomal membrane representing the absolute host–symbiont interface. The symbiosomal membrane must be a dynamic and selectively permeable structure to enable bidirectional and differential movement of essential nutrients, metabolites, and biosynthetic intermediates, vital for growth and survival of host and symbiont. However, despite this crucial role, the molecular basis of membrane transport across the symbiosomal membrane remains unresolved in all bacteriocyte-containing insects. A transport protein was immunolocalized to the symbiosomal membrane separating the pea aphid *Acyrthosiphon pisum* from its intracellular symbiont *Buchnera aphidicola*. The transporter, *A. pisum* nonessential amino acid transporter 1, or ApNEAAT1 (gene: *ACYPI008971*), was characterized functionally following heterologous expression in *Xenopus* oocytes, and mediates both inward and outward transport of small dipolar amino acids (serine, proline, cysteine, alanine, glycine). Electroneutral ApNEAAT1 transport is driven by amino acid concentration gradients and is not coupled to transmembrane ion gradients. Previous metabolite profiling of hemolymph and bacteriocyte, alongside metabolic pathway analysis in host and symbiont, enable prediction of a physiological role for ApNEAAT1 in bidirectional host–symbiont amino acid transfer, supplying both host and symbiont with indispensable nutrients and biosynthetic precursors to facilitate metabolic complementarity.

## Significance

Plant sap-feeding insects thrive despite feeding exclusively on a diet lacking in essential amino acids. This nutritional deficit is countered through endosymbiotic relationships with microbial symbionts. Nonessential amino acids, vital for microbial symbionts, are utilized by symbiont metabolic pathways and yield essential amino acids required by their eukaryotic hosts. Symbionts are completely dependent on their host to meet nutritional requirements. The endosymbionts are surrounded individually by host-derived symbiosomal membranes and are housed within specialized host bacteriocyte cells. The transport capabilities of the symbiosomal membrane remain unknown. Here, we identify a transport system that mediates a crucial step in this metabolic complementarity: a transporter capable of transporting nonessential amino acids across the symbiosomal membrane of the pea aphid *Acyrthosiphon pisum*.

Animals and plants live in symbiosis with a complex microbiota. Such symbioses are ubiquitous and impact the biology of all multicellular organisms (1–3). While symbioses are pervasive, the cellular and molecular mechanisms that function at the interface of hosts and symbionts remain largely unknown. One particularly intriguing and intimate type of symbiotic interaction is endosymbiosis, involving one partner, the symbiont, living inside the cells of the other partner, the host. Endosymbiotic partnerships are prevalent in groups of insects that feed on plant sap and vertebrate blood (4–8).

**Fig. 1.**
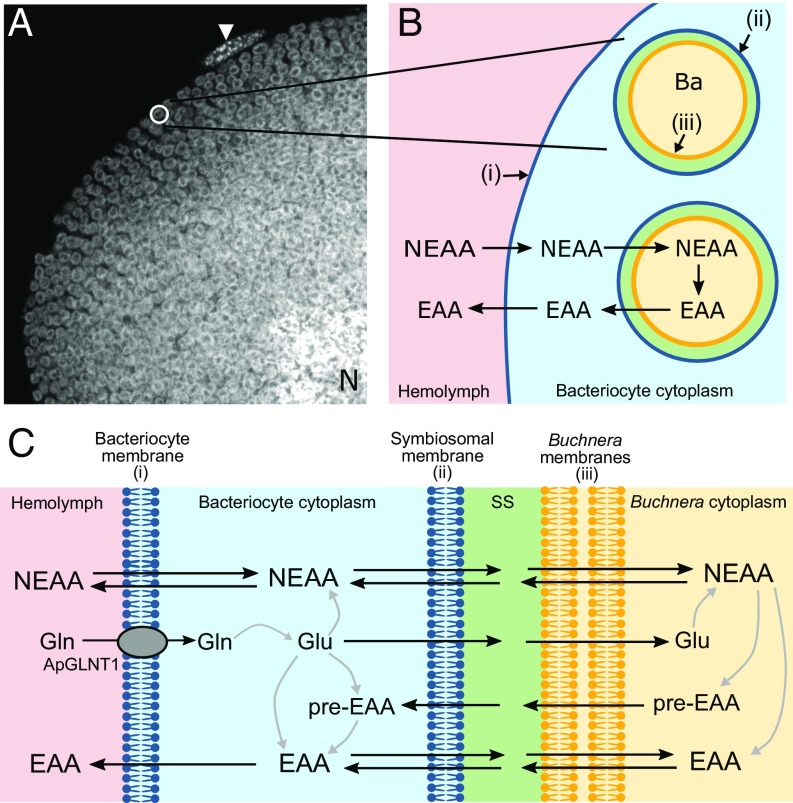
The aphid/*Buchnera* symbiotic boundary and the role in amino acid exchange. (*A*) *A. pisum* bacteriocytes each harbor thousands of bacterial endosymbionts (*Buchnera aphidicola*). A greyscale confocal (magnification: 630×) image showing DAPI-associated fluorescence (identifying nuclear and *Buchnera* DNA) through an *A. pisum* bacteriocyte packed full with *Buchnera* endosymbionts (visualized by their typical spherical shape, 3 μm in diameter). N represents host nucleus. The arrowhead indicates a sheath cell on the bacteriocyte periphery. (*B*) Schematic representation of the aphid/*Buchnera* boundary highlighting the endosymbiotic paradigm, where the host supplies symbiont with NEAAs and the symbiont provides host with EAAs. A series of membranes separate the hemolymph from the symbiont: (*i*) the aphid (host) bacteriocyte membrane (blue) separates hemolymph from bacteriocyte cytosol; (*ii*) the host-derived symbiosomal membrane (blue) separates each individual *Buchnera* from the bacteriocyte cytosol; (*iii*) the outer and inner membranes (yellow) of the gram-negative *Buchnera*. Ba, *B. aphidicola*. (*C*) More detailed schematic representation of the putative steps in NEAA and EAA transport across the aphid/*Buchnera* symbiotic boundary. The only identified amino acid transporter to date is the glutamine-selective ApGLNT1, which is localized in the bacteriocyte membrane (28). Much of the glutamine taken into the bacteriocyte is converted into glutamate, which can either be transported across the symbiosomal membrane or converted by bacteriocyte enzymes into NEAAs. The NEAAs must cross the symbiosomal membrane to be utilized by *Buchnera* and in the *Buchnera*-mediated production of other NEAAs, EAAs, or EAA precursors (pre-EAA), all of which can exit across the symbiosomal membrane back into the bacteriocyte cytosol. SS, symbiosomal space.

The insect order Hemiptera, including aphids, mealybugs, and whiteflies, is highly successful and widespread despite feeding exclusively on nutrient-deficient plant sap (9–11). To enable optimal utilization of diet, sap-feeding Hemipterans exist in a state of endosymbiosis with microbial symbionts (4, 7, 12). The ancient cooperation between the pea aphid *Acyrthosiphon pisum* and its intracellular symbiont *Buchnera aphidicola* is an exemplar insect endosymbiosis, being obligate and mutualistic, with each partner required for survival and reproduction of the other (7, 13–17). The symbiont is located within specialized insect cells, called bacteriocytes (Fig. 1*A*), in a larger organ-like structure, known as the bacteriome, that lines the abdomen and surrounds the aphid gut (4, 7, 16, 18, 19). Endosymbiont-containing bacteriocytes are found in up to 20% of all insect species (14). The boundary between aphid and *Buchnera* (Fig. 1 *B* and *C*) exists as a series of membrane barriers: (*i*) the bacteriocyte cell membrane (separating hemolymph from bacteriocyte cytosol); (*ii*) the aphid-derived symbiosomal membrane (surrounding individual symbionts, enabling separation from bacteriocyte cytosol); and (*iii*) the outer and inner membranes of *Buchnera*. The symbiosomal membrane defines the absolute host–symbiont interface (Fig. 1 *B* and *C*).

The eukaryotic host and prokaryotic symbiont exist in an interdependent state of complementary nutritional and metabolic symbiosis (20). Metazoans are unable to synthesize certain amino acids in quantities to satisfy growth and development and these essential amino acids (EAAs) are obtained usually from diet. However, phloem sap is a particularly poor source of EAAs but is relatively rich in other nonessential amino acids (NEAAs) (9–11). The microbial symbiont lacks most NEAA biosynthetic pathways but possesses many key components in EAA synthesis (15, 16, 20, 21). A paradigm has evolved where the insect is considered to supply the symbiont with NEAAs and, in return, the symbiont provides the insect with EAAs, or critical biosynthetic pathway components (Fig. 1*B*). Although correct in general terms, it is evident that individual biosynthetic pathways for NEAAs and EAAs are not partitioned exclusively to either the host or symbiont. Rather, the relationship is complex and integrated with key biosynthetic steps in single pathways being encoded by a combination of endosymbiont and host genomes (13–16, 20–27) (Fig. 1*C*).

While the logic behind the foundation of the nutritional collaboration is well-defined, the molecular interdependence of the endosymbiotic relationship remains something of a black box (28). For net movement of nutrients, metabolites, and biosynthetic intermediates to occur across the host/symbiont boundary (Fig. 1*C*), the symbiosis is dependent exquisitely upon each membrane expressing a unique repertoire of transport proteins to enable movement in one direction or the other, as required. In particular, understanding the dynamic function of the symbiosomal membrane is crucial for uncovering the role of this symbiont–host interface in the success of each symbiosis and the wider Hemipteran order. How is material transferred across the symbiosomal membrane from host to symbiont, and vice versa, in aphids or any other insect?

The transport of NEAAs across the symbiosomal membrane is absolutely key to the success of the symbiosis to support growth and development of the symbiont and to provide biosynthetic precursors to enable synthesis and supply of EAAs to the host. The current investigation had one primary objective: to identify the amino acid transporter involved in NEAA transport across the absolute host–symbiont interface, the symbiosomal membrane (Fig. 1 *B* and *C*). To achieve that goal, an integrated computational and experimental approach was utilized. A prime candidate amino acid transporter (ACYPI008971) was identified from the pea aphid *A. pisum* based on gene expression, sequence alignment, and prediction of 3D structure. The amino acid transport protein was localized to the *A. pisum* symbiosomal membrane by immunocytochemistry. The functional characteristics of this transport system were determined following heterologous expression in *Xenopus laevis* oocytes, resulting in the transporter being named *A. pisum* nonessential amino acid transporter 1 (ApNEAAT1). Elucidation of the functional characteristics of ApNEAAT1 transport enables prediction of the likely fundamental role played by this carrier in bidirectional amino acid transfer between host and symbiont, and thus the success of the symbiosis as a whole.

## Results

### Identification of the Candidate Symbiosomal Amino Acid Transporter ApNEAAT1 (ACYPI008971).

Bacteriocytes (Fig. 1*A*) function as specialized amino acid-producing factories with aphid and *Buchnera* cooperating to synthesize a complete gamut of amino acids (20). To produce this single integrated metabolic network (20), the symbiosomal membrane (Fig. 1*C*) must allow the selective exchange of amino acids and intermediates between compartments to supply each enzymatic step in each compartment. The molecular mechanisms responsible for such transmembrane exchanges are unknown. However, candidate transporters have been identified from transcript information, expression of some being up-regulated in bacteriocytes (20, 24, 28–31). Interrogation of the *A. pisum* and *Buchnera* genomes, and measurements of metabolic pathway gene and protein expression in bacteriocyte tissues, have enabled the direction of net flow of individual NEAAs and EAAs to be predicted (16, 20, 23, 29, 32–35). Net movement of individual amino acids will be controlled, to an extent, by the expression and substrate specificity of the amino acid transporters at each of the membranes (Fig. 1). Some NEAAs (e.g., glutamine, asparagine, and glutamate) are abundant in hemolymph and bacteriocyte cytosol. Therefore, effective transfer of less abundant NEAAs (e.g., proline, alanine, glycine, serine, and cysteine) is likely via a separate carrier that will exclude those abundant NEAAs to avoid competition.

**Fig. 2.**
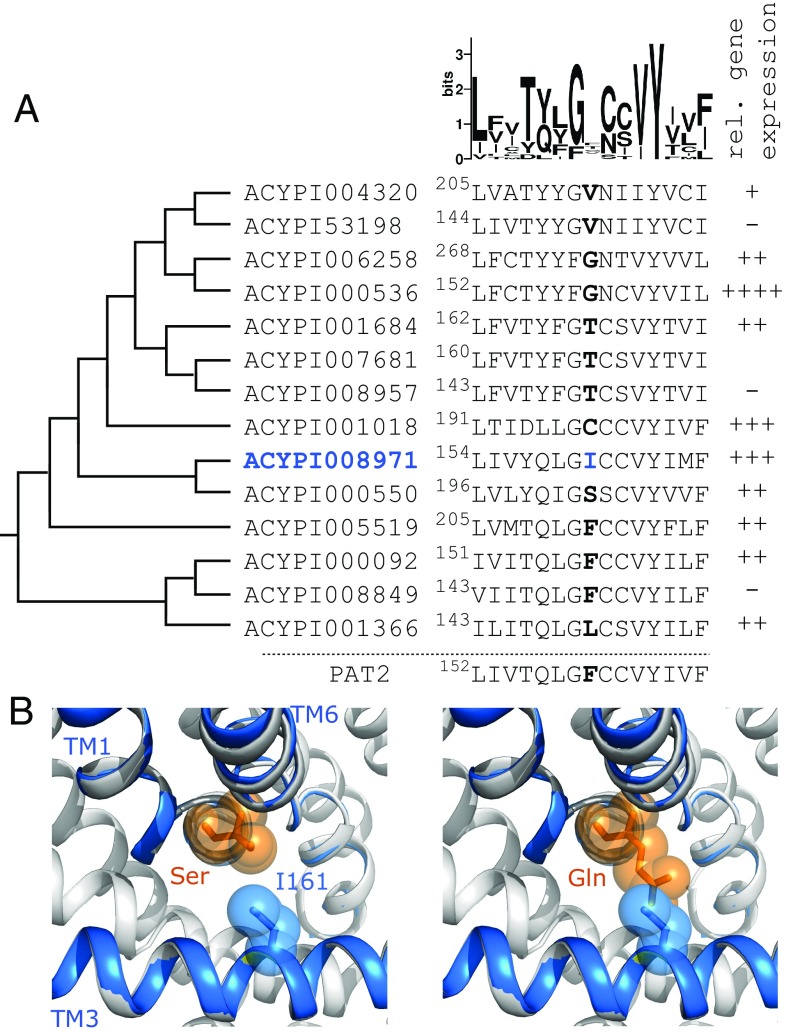
Identification of aphid ApNEAAT1 (ACYPI008971) as a putative carrier of small NEAAs expressed in the bacteriocyte. (*A*) *Left* column: phylogeny showing the relationship between all 14 SLC36-related *A. pisum* AAAP transporters from the arthropod expanded AAAP clade (30, 31). Phylogenetic tree based on previously published phylogenies (30, 50). *Middle* column: portion of a full sequence alignment (by PROMALS3D) showing the central section of TM3 with a representation of the variability at each residue position shown above as a Sequence Logo. The residues highlighted in bold are equivalent to both F159 in rat PAT2 (slc36a2) and V104 in LeuT (39, 51). ACYPI008971 has I161 (blue) at this residue position. *Right* column: Representation of relative gene expression of each transporter within the bacteriocyte structure as a whole. −, not expressed; ++++, most highly expressed; +++, ≤35%; ++, ≤15%, +, ≤1% expression of the most highly expressed amino acid transporter [summary of gene expression determined by RNAseq which is consistent with earlier estimates using qPCR (28, 30)]. ACYPI007681 expression was not determined. (*B*) A structural model of ACYPI008971 was created using I-TASSER and aligned against the highest-scoring crystal (3L1L of the arginine transporter AdiC, gray). Sections of ACYPI008971 TM1, TM3, and TM6 are shown as blue ribbons. ACYPI008971 I161 (blue sticks and spheres) projects toward the substrate binding pocket. When serine or glutamine (orange sticks and spheres) were positioned in the binding pocket, using the arginine in the 3L1L crystal as a guide, it shows that I161 is likely to limit binding pocket space so that ACYPI008971 may transport amino acids with shorter (Ser) rather than longer (Gln) side-chains.

The Transporter Classification Database (36) groups the largest collection of amino acid transporters across all forms of life within the amino acid-polyamine-organocation (APC) superfamily (37). Within the APC superfamily, many eukaryotic amino acid transporters are grouped within the important amino acid/auxin permease (AAAP, TC# 2.A.18) family, expressed ubiquitously in animals, fungi, yeast, and plants (37–39). In mammals, 4 members of the AAAP family are found within the solute carrier (SLC) family SLC36 (38). Mammalian PAT1 (SLC36A1) and PAT2 (SLC36A2) are important in transmembrane transport of the small NEAAs proline, alanine, and glycine in neural, intestinal, and renal tissues across both the plasma membrane and intracellular organelles (40–48). The SLC36 family is evolutionarily very old and likely had a single founding member conserved through evolution with duplications before the teleost lineage, before the separation of reptiles and birds, and a third, which is probably mammalian-specific (49). In invertebrates, this SLC36-related AAAP family has undergone extensive expansion with duplications in the common arthropod ancestor and more recent aphid-, psyllid-, whitefly-, and mealybug-specific expansions in Hemiptera (30, 31, 39, 50). In *A. pisum*, there are 14 putative, SLC36-related AAAP transporters (Fig. 2*A*). Several are highly expressed and highly enriched in bacteriocytes (28–31, 50). An RNA sequencing (RNA-seq) estimation of the relative expression of these 14 AAAP transporter-related genes [consistent with qPCR measurements across several *A. pisum* lines (28, 30)] demonstrates variable expression in total bacteriocyte tissue. High gene expression is observed for *ACYPI00536*, *ACYPI001018* (*A. pisum* glutamine transporter 1, ApGLNT1) and *ACYPI008971* (Fig. 2*A*).

Transporters within the AAAP family are predicted to have a 3D structure (known as the LeuT-fold) consisting of a 10-transmembrane (TM) core organized into a 5 + 5 inverted structural repeat (51). The substrate binding pocket of the carrier is formed by TM1, TM3, TM6, and TM8 (51, 52). Recently, we determined the importance of a single position in TM3 of the LeuT-fold in AAAP transporters, with the residue in that position shaping the bottom of the hydrophobic substrate binding pocket into which the substrate side-chain fits (39). The size of the residue occupying that single position in TM3, in exemplar mammalian and arthropod AAAP amino acid transporters, determines substrate selectivity by limiting the space available for the amino acid substrate side-chain (39). For example, the mammalian amino acid transporter PAT2 (SLC36A2) has a large aromatic phenylalanine at this position, which severely restricts the accessible space within the binding pocket and limits substrate selectivity to proline, alanine, and glycine (39). Replacement of phenylalanine (191.9 Å^3^) in the substrate binding pocket with the smaller branched side-chain of isoleucine (163.9 Å^3^) (53) increases the accessible space and creates the PAT2-F159I gain-of-function mutant (39). In addition to proline, alanine, and glycine, PAT2-F159I transports serine and cysteine but excludes amino acids with larger side-chains, such as glutamine, asparagine, and glutamate (39). Application of that observation, suggests that an *A. pisum* SLC36-like AAAP transporter with an isoleucine residue at that key position in TM3 would most likely be a transporter of serine, proline, alanine, cysteine, and glycine, as observed with PAT2-F159I (39). The 14 *A. pisum* SLC36-like AAAP transporters were multialigned with rat PAT2 using PROMALS3D (54). Fig. 2*A* shows part of TM3. From the sequence logo (55) in Fig. 2*A*, it is clear that only 1 position in TM3 in these putative aphid AAAP carriers is completely conserved [a tyrosine, being equivalent to LeuT Y108, which forms part of the hatch in the outward-occluded substrate-bound LeuT crystal (51)]. In contrast, the residues equivalent to PAT2 F159 (those highlighted in bold in Fig. 2*A*) show the greatest variability in this section of TM3, consistent with this position being important in determining variable substrate selectivity across this group of carriers.

ACYPI008971 is the only *A. pisum* SLC36-like carrier to have an isoleucine residue (I161) at the position equivalent to PAT2 F159 (39). Models of both ACYPI008971 and PAT2 were generated using I-TASSER (56). When both models were superposed upon the highest scoring APC superfamily/LeuT-fold crystal [the outward-occluded, arginine-bound AdiC crystal (3L1L) (52)], the predicted position for ACYPI008971 I161 and PAT2 F159 overlapped (*SI Appendix*, Fig. S1), which was consistent with predictions using HHPred/Modeller (39, 57, 58) and PROMALS3D (54) (Fig. 2*A*). The small, zwitterionic, NEAA serine was superposed upon the arginine backbone (within the binding pocket of the AdiC crystal, 3L1L) (Fig. 2*B*). The model shows that serine is predicted to fit within the binding pocket and is presumably transported, whereas the longer side-chain of the NEAA glutamine clashes with ACYPI008971 I161, suggesting that glutamine will be excluded and not transported by ACYPI008971 (Fig. 2*B*) as observed with PAT2-F159I (39).

Two other SLC36-related AAAP transporters are highly expressed in bacteriocyte tissues (Fig. 2*A*). ApGLNT1 (ACYPI001018) is expressed at the bacteriocyte, but not symbiosomal, membrane (28). ApGLNT1 has a cysteine (C198) at the position equivalent to PAT2 F159 (Fig. 2*A*). The smaller cysteine (103.3 Å^3^) (53) suggests that there is greater accessible space within the ApGLNT1 binding pocket, suitable for transport of amino acids with longer side-chains. Indeed, ApGLNT1 is highly selective for the larger NEAA glutamine (with arginine being a nontransported inhibitor) but does not transport smaller NEAAs, such as proline, serine, alanine, cysteine, and glycine (28). ACYPI00536 has a glycine (G159) at the position equivalent to PAT2 F159 (Fig. 2*A*). The predicted accessible space within the ACYPI00536 binding pocket suggests that it would not favor small NEAAs as substrates (this has been observed in other AAAP transporters as the binding pocket space is increased) (39) but would more likely transport amino acids with much longer side-chains.

Thus, based upon the predicted structure and key binding pocket residue, combined with high expression in bacteriocyte tissue, ACYPI008971, an uncharacterized, putative transport protein, was identified as the prime candidate to be the symbiosomal small NEAA transporter, fundamental to the symbiosis as a whole, where it will mediate selective movement of small NEAAs across the aphid/*Buchnera* symbiotic interface. Based upon this predicted function, the transporter is henceforth referred to as ApNEAAT1.

### Immunolocalization of ApNEAAT1 at the Symbiosomal and Bacteriocyte Membranes.

**Fig. 3.**
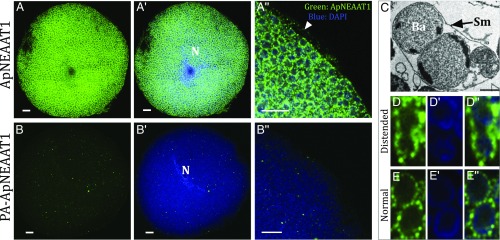
Immunolocalization of ApNEAAT1 to the symbiosomal and bacteriocyte membranes of isolated bacteriocyte cells. (*A*) Immunolocalization of ApNEAAT1 (green) reveals extensive punctate staining around individual *Buchnera* cells. (*A*′) Merge of the anti-ApNEAAT1 image and DAPI-stained nuclear and *Buchnera* DNA (blue). (*A*″) Magnified region of bacteriocyte cell showing merge of anti-ApNEAAT1 localization (green) and DAPI-stained DNA (blue), arrowhead marks localization to the bacteriocyte cell membrane. (Scale bars, 10 μm.) (*B*–*B*″) Comparable control experiments were performed with isolated *A. pisum* bacteriocytes with peptide preadsorbed (PA) anti-ApNEAAT1 antibody. The secondary antibody was Alexa-Fluor 568 donkey anti-rabbit IgG (H+L) (Scale bars, 10 μm). N, bacteriocyte cell nucleus. (*C*) TEM of distended symbiosomal membrane (Sm) enclosing 2 *B. aphidicola* (Ba). (Reprinted from ref. 59, with permission from Elsevier.) Left to right: (*D*) Immunolocalization of ApNEAAT1 to the distended symbiosomal membrane; (*D*′) DAPI-stained *Buchnera* cells; (*D*″) merge of the anti-ApNEAAT1 image (green) and DAPI-stained *Buchnera* DNA (blue). (*E*–*E*″) Comparable images of 2 *Buchnera* surrounded by their own symbiosomal membranes. For all images, a single representative confocal plane is shown for 3 replicated localization experiments (*SI Appendix*, Fig. S2).

To date, no transport protein has been immunolocalized to the symbiosomal membrane in any insect. Immunolocalization of ApNEAAT1 protein to the *A. pisum* bacteriocyte using an anti-ApNEAAT1 antibody reveals abundant expression of ApNEAAT1 throughout the bacteriocyte (Fig. 3). Extensive punctate staining (green) is evident surrounding each of the densely packed *Buchnera* cells (Fig. 3 *A*–*A*″). Staining was absent in control panels performed with either peptide-preadsorbed primary anti-ApNEAAT1 antibody (Fig. 3 *B*–*B*″) or secondary-only antibodies (*SI Appendix*, Fig. S2), confirming the specificity of the ApNEAAT1 immunolocalization. Identical localization patterns were consistent in 3 independent experiments (*SI Appendix*, Fig. S2). Nuclei and *Buchnera* are identified by DAPI (blue) (Fig. 3). Normally, each individual *Buchnera* cell is surrounded by its own symbiosomal membrane. However, the symbiosomal membrane is a dynamic structure that undergoes fission events to accommodate the growth and propagation of the bacterial symbiont (15, 19). During cell division, the symbiosomal membrane becomes stretched and 2 *Buchnera* can be observed within a single extended symbiosomal compartment (as shown in the transmission electron microscope [TEM] image in Fig. 3*C*) (18, 19, 59). The continuous punctate staining on the distended symbiosomal membrane enclosing 2 *Buchnera* demonstrates that ApNEAAT1 is localized to the symbiosomal membrane and not *Buchnera* cell membranes (Fig. 3*D*). Furthermore, the immunolocalization of ApNEAAT1 also reveals punctate staining on the bacteriocyte plasma membrane (Fig. 3*A*″), demonstrating that the ApNEAAT1 transport protein is expressed at both the symbiosomal and bacteriocyte membranes within the *A. pisum*/*Buchnera* symbiotic boundary, where we predict it plays an essential role in amino acid movement between key compartments in the endosymbiotic structure.

### ApNEAAT1 Is a Transporter of the Small Dipolar NEAAs Proline, Alanine, Serine, Cysteine, and Glycine.

**Fig. 4.**
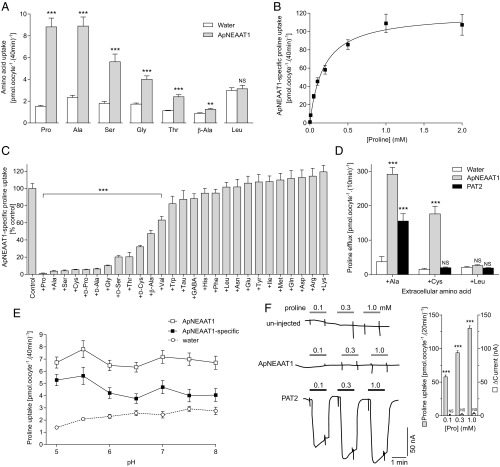
The aphid amino acid carrier ApNEAAT1 transports the NEAAs proline, serine, alanine, glycine, and cysteine. (*A*) Uptake of various radiolabeled amino acids (10 µM) into ApNEAAT1-expressing and water-injected (control) oocytes measured in the absence of extracellular Na^+^ at pH 5.5. *n* = 20. ***P < 0.001; **P < 0.01; NS (not significant), *P* > 0.05 vs. water (2-way, unpaired *t* tests). (*B*) ApNEAAT1-specific, concentration-dependent proline uptake. Uptake into water-injected oocytes was subtracted from total uptake. Curve is fitted to Michaelis–Menten kinetics [*K*_m_ = 179 ± 33 µM; *V*_max_ = 120 ± 6 pmol.oocyte^−1^.(40 min)^−1^; *r*^2^ = 0.986]. *n* = 20. (*C*) Proline uptake in the absence (control) and presence of amino acids or analogs (all 10 mM except Tyr which is 2.5 mM). All are l-isomers unless indicated otherwise. ApNEAAT1-specific uptake is calculated by subtraction of uptake into water-injected oocytes and is expressed as percent control (that in the absence of inhibitor). Tau, taurine. *n* = 16–20. ***P < 0.001 vs. control; all other bars are *P* > 0.05 vs. control (1-way ANOVA with Sidak’s posttest). (*D*) Trans-stimulation of proline ([5 mM]_i_) efflux via ApNEAAT1 and PAT2 (rat slc36a2) by various extracellular amino acids (10 mM) was measured under Na^+^-free conditions at extracellular pH 5.5 (10 min). *n* = 4–5. ***P < 0.001; NS, *P* > 0.05 vs. water (2-way ANOVA with Tukey’s posttest). (*E*) Proline uptake in Na^+^-free conditions over the pH range 5.0–8.0. *n* = 20. The only significant difference found within each group was in ApNEAAT1-specific uptake: pH 6.5 vs. pH 5.5, *P* = 0.046 (2-way ANOVA with Tukey’s posttest). (*F*) Proline-associated inward current in PAT2-expressing but not ApNEAAT1-expressing or uninjected (control) oocytes as measured by 2-electrode voltage clamp. Oocytes were clamped at resting V_M_ (−30 mV), superfused with Na^+^-free, pH 5.5 buffer and exposed to proline (0.1 to 1 mM). Mean data are shown in (*SI Appendix*, Table S1) and for ApNEAAT1 in the *Inset*. (*Inset*) As a direct comparison, proline uptake via ApNEAAT1 was measured under the same conditions as current measurement. ***P < 0.001.

When expressed in *X. laevis* oocytes, as predicted, ApNEAAT1 transports dipolar NEAAs, with relatively small side-chains, such as proline, alanine, serine, and glycine (Fig. 4*A*). ApNEAAT1 is saturable, with a relatively high affinity (proline uptake, *K*_m_ = 179 ± 33 µM) (Fig. 4*B*). Competition experiments (Fig. 4*C*) complement the uptake measurements (Fig. 4*A*) and suggest that ApNEAAT1 substrates include a broad range of the smaller dipolar l- and d-amino acids (including proline, alanine, serine, cysteine, and glycine) but also weaker interactions with amino acids of slightly larger side-chain (e.g., threonine) or the straight-chain amino acid β-alanine (Fig. 4 *A* and *C*). Amino acids with even larger side-chains are excluded (Fig. 4 *A* and *C*). Importantly, ApNEAAT1 avoids unnecessary competition between its substrates and other amino acids by excluding those abundant in phloem and hemolymph (glutamine and asparagine) and those, such as glutamate, synthesized at high levels in the bacteriocyte, all of which have no significant (*P* > 0.05) effect on ApNEAAT1-mediated proline uptake (Fig. 4*C*). ApNEAAT1 can transport amino acids in either inward or outward directions (Fig. 4*D*). [^3^H]Proline efflux was limited from control (water-injected) oocytes under all conditions (Fig. 4*D*). However, in ApNEAAT1-expressing oocytes, [^3^H]proline efflux was markedly increased in the presence of the extracellular substrates alanine or cysteine but not in the presence of the nonsubstrate leucine (Fig. 4*D*). The transstimulation is observed because alanine and cysteine are transported into the cell by ApNEAAT1, which increases the availability of the transporter for [^3^H]proline efflux. Generally, fully loaded carriers move through their transport cycles more rapidly than empty carriers (60, 61). Cysteine is thus demonstrated to be an ApNEAAT1 substrate by its ability to trans-stimulate [^3^H]proline efflux (Fig. 4*D*). In PAT2-expressing oocytes, alanine trans-stimulation of [^3^H]proline efflux was evident but, in contrast to ApNEAAT1, there was no trans-stimulation by cysteine (Fig. 4*D*) (consistent with cysteine and leucine not being substrates for wild-type PAT2) (44, 62, 63).

Unlike other mammalian and arthropod SLC36-related AAAP transporters characterized to date (mammalian PAT1 and PAT2, and the arthropod carriers *A. pisum* ApGLNT1, *Aedes aegypti* AaePAT1, and *Drosophila melanogaster* CG1139) (36, 28, 39, 42, 63, 64), ApNEAAT1-mediated amino acid transport is not driven by the H^+^-electrochemical gradient, as demonstrated by the lack of pH dependence over pH range 5.0 to 9.0 (Fig. 4*E* and *SI Appendix*, Fig. S3). ApNEAAT1-mediated amino acid transport is independent of ionic gradients for H^+^, Na^+^, K^+^, and Cl^−^ (Fig. 4*E* and *SI Appendix*, Fig. S3). The mammalian PAT1 (SLC36A1) and PAT2 (SLC36A2), transporters both function as H^+^/amino acid cotransporters with 1:1 stoichiometry (42, 65). The protonophore FCCP diminishes the H^+^-electrochemical gradient and reduces H^+^/amino acid cotransport via PAT1 but has no effect on ApNEAAT1-mediated amino acid transport (*SI Appendix*, Fig. S3). H^+^/amino acid symport by PAT2 is associated with inward, amino acid-coupled, H^+^ transport in voltage-clamped *Xenopus* oocytes (as demonstrated by the downward deflection of the trace during exposure to extracellular proline in Fig. 4*F*). In contrast, no inward currents were detected in control (uninjected) or ApNEAAT1-injected oocytes even though ApNEAAT1-mediated, concentration-dependent, [^3^H]proline uptake was observed in parallel experiments performed under the same conditions (Fig. 4*F*). Thus, in contrast to PAT1 and PAT2, both of which are H^+^/amino acid symporters, ApNEAAT1 transports amino acids by a mechanism that is not dependent on extracellular pH, is not rheogenic, and is not driven by the H^+^-electrochemical gradient (Fig. 4 *E* and *F* and *SI Appendix*, Fig. S3). Interestingly, when voltage-clamped at more hyperpolarized membrane potentials, small, poorly reversing, inward deflections during exposure to saturating ApNEAAT1 substrate concentrations were observed (*SI Appendix*, Fig. S4 and Table S1). These were disproportionally small, relative to amino acid transport, and perhaps represent nonstoichiometric slippage currents described in other members of the AAAP transporter family and wider APC superfamily (66–69).

**Fig. 5.**
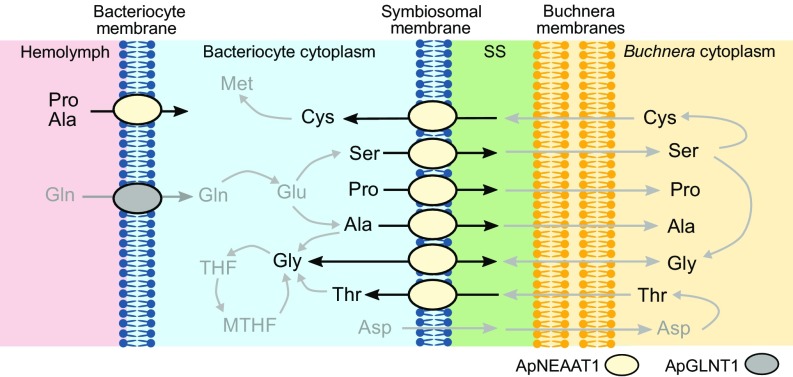
A schematic model depicting the proposed physiological function of the amino acid transporter ApNEAAT1 in amino acid transfer across the bacteriocyte and symbiosomal membranes in the aphid/*Buchnera* bacteriocyte. The predicted pathways are based on the membrane localization and functional characterization of ApNEAAT1 here, alongside metabolite profiling of hemolymph and bacteriocyte, and host and symbiont metabolic pathway analysis (protein and gene expression) in previous investigations (16, 20, 21, 24, 29, 32–35). ApNEAAT1 substrates are identified in black text with nonsubstrates presented in gray text. The bacteriocyte membrane ApNEAAT1 is depicted as mediating influx into the bacteriocyte. However, if the concentration of any ApNEAAT1 substrate within the bacteriocyte was greater than in the hemolymph, it could also mediate efflux from bacteriocyte to hemolymph to supply other tissues, for example, during growth and development. MTHF, 5,10-methylene tetrahydrofolate; SS, symbiosomal space.

In summary, ApNEAAT1 is an amino acid transport system localized to both the symbiosomal and bacteriocyte membranes (Fig. 3). ApNEAAT1 is an electroneutral transporter of small NEAAs (such as glycine and l- and d-proline, serine, alanine, and cysteine) capable of transport in both inward and outward directions (Fig. 4). The membrane localization and functional characteristics of ApNEAAT1 enable the prediction that ApNEAAT1 will mediate amino acid transport across the symbiosomal membrane in both host-to-symbiont and symbiont-to-host directions (Fig. 5), with net transport of any particular amino acid driven by local transmembrane amino acid concentration gradients. We propose that the transporter be named ApNEAAT1 to reflect its origin and primary function.

## Discussion

Life forms have evolved to occupy unique environmental niches. The ability of eukaryote and microbial endosymbiotic partnerships, in both animal and plant hosts, to exploit such habitats reflects a triumph of cooperation, coordination, and compartmentalization. Metabolic cooperation, the complementation of pathways using genes encoded in host and symbiont genomes, is a signature of host/symbiont coevolution (70). The small, highly reduced genome of *Buchnera* retains genes for the biosynthesis of 13 amino acids and some B vitamins, nutrients that are in short dietary supply (32, 71). Remarkably, the biosynthesis of many nutrients provisioned to the aphid requires complementation of *Buchnera* metabolic pathways by enzymes encoded in the host genome (23, 33). The evolution of such metabolic complementarity occurs across a breadth of diverse insect species in a handful of metabolic pathways, the most notable including the branched-chain amino acids (21, 25–27, 29) and the B vitamin pantothenate (70, 72). The need for nutrient and metabolite transport across the endosymbiotic membranes is absolute. However, except for a glutamine-specific transporter (ApGLNT1) localized to the bacteriocyte membrane (28), the roles of transporters in mediating and controlling these endosymbiotic nutrient movements remain a mystery.

The symbiosomal membrane forms a physical barrier that separates the 2 halves of this integrated metabolic network. However, it is not an impenetrable impediment to free movement between the 2 compartments but rather a dynamic and selectively permeable structure that enables bidirectional movement of nutrients, metabolites, and biosynthetic intermediates between organisms. The transport mechanisms that reside within the symbiosomal membrane remain unidentified, in any insect, and their functional capabilities uncharacterized. ApNEAAT1 was localized to both the symbiosomal (confirmed by the immunocytochemical pattern observed in the extended symbiosomes) and bacteriocyte membranes (Fig. 3). This pattern is consistent with quantitative proteomic analysis that identified ApNEAAT1 protein in the bacteriocyte-residual fraction (the bacteriocyte fraction lacking *Buchnera*) but not in the proteome recovered from isolated *Buchnera* (29).

Functional prediction using homology modeling identified ApNEAAT1 (gene name: *ACYPI008971*) as a candidate for the small NEAA transport that is necessary at the symbiosomal membrane (Fig. 2). The functional characterization of ApNEAAT1 in *Xenopus* oocytes demonstrates that it is an amino acid transport system with a preference for the small dipolar NEAAs proline, serine, alanine, cysteine, and glycine but excludes amino acids with larger side-chains, such as asparagine, glutamine, and glutamate (Fig. 4). This electroneutral transporter can work in both inward and outward directions and is driven by prevailing amino acid concentration gradients rather than ionic gradients (Fig. 4), making it an ideal portal for bidirectional movement of amino acids across membrane barriers (Fig. 5). What physiological and endosymbiotic roles might ApNEAAT1 perform?

At the symbiosomal membrane, there is a requirement for inward (bacteriocyte-to-symbiont) movement of serine, proline, and alanine, potential bidirectional movement of glycine, and symbiont-to-bacteriocyte efflux of cysteine (Fig. 5). *Buchnera* possess most enzymes for synthesis of EAAs, but those for synthesis of 7 NEAAs (including serine, alanine, and proline) are absent (20, 23, 29, 33). Aphid genes involved in biosynthesis of 5 of the NEAAs (including serine and alanine), not synthesized by *Buchnera*, are up-regulated in the bacteriocyte (relative to aphid body) (20). Genes involved in proline biosynthesis are highly expressed in both aphid body and bacteriocyte, indicating that proline is synthesized at high levels in the host as a whole (20). Thus, ApNEAAT1 could transport host-synthesized serine, alanine, and proline from the bacteriocyte to *Buchnera* to be utilized directly (e.g., protein synthesis) or in further symbiont metabolic pathways. For example, serine is 1 of the 4 NEAAs required by *Buchnera* as an amino group donor (20, 23, 29) for synthesis of host-required EAAs, such as methionine (16, 20). Serine must also be transported into *Buchnera* for conversion into 2 other NEAAs, cysteine and glycine (20, 29), which are utilized directly by *Buchnera* but which could also be effluxed back into the bacteriocyte (20, 23, 24), a role that ApNEAAT1 could fufill. Indeed, the predicted flux of cysteine across the symbiosomal membrane is in the symbiont-to-bacteriocyte direction where it is anticipated to lead to bacteriocyte-mediated homocysteine production followed by synthesis of the EAA methionine in host or symbiont (Fig. 5) (20). Thus, a key role for ApNEAAT1 might be in enabling metabolic complementarity where host-derived serine is transported across the symbiosomal membrane to symbiont, converted to cysteine, and returned (via ApNEAAT1-mediated symbiosomal transport) to the bacteriocyte for the final stages of methionine synthesis (Fig. 5) (16, 20, 33). Similarly, *Buchnera*-derived glycine is predicted to efflux across the symbiosomal membrane (24) to be a cofactor in bacteriocyte conversion of THF into 5,10-methylene THF (20, 35). Threonine is synthesized within *Buchnera* from host-derived aspartate and is predicted to exit the symbiont to be utilized in glycine biosynthesis within the bacteriocyte cytosol (16, 20, 24). Although threonine is transported relatively poorly by ApNEAAT1 (Fig. 4), it could still mediate symbiont-to-host threonine transfer (Fig. 5).

The second key role of ApNEAAT1 within the endosymbiotic organ will occur at the bacteriocyte membrane (Fig. 5). Metabolite profiling of hemolymph enables metabolic modeling of the likely flux of amino acids across the bacteriocyte membrane into the bacteriocyte (13, 24). The predicted flux estimates suggest that the 4 major amino acid fluxes into the bacteriocyte are asparagine (51.6 units), glutamine (16.2 units), proline (6.5 units), and alanine (4.9 units) (24). Proline and alanine influx could be mediated via ApNEAAT1. The exclusion of asparagine and glutamine by ApNEAAT1 is crucial as they are the 2 most abundant amino acids in hemolymph and would, if transported by ApNEAAT1, create unnecessary competition for proline and alanine transport (13). In addition, the bacteriocyte, functioning as an amino acid biosynthetic factory, could generate NEAA concentrations that are higher than those in hemolymph. Under those circumstances, ApNEAAT1-mediated amino acid efflux across the bacteriocyte membrane could support other processes, for example, during embryogenesis.

These observations suggest that ApNEAAT1 has dual roles in amino acid transport at 2 key sites within the endosymbiosis mediating bidirectional amino acid transport across the bacteriocyte (between hemolymph and bacteriocyte) and symbiosomal (between bacteriocyte and symbiont) membranes (Fig. 5). The one-to-one orthology of ApNEAAT1 and related orthologs across many Hemipteran species (including aphids, psyllids, mealybugs, and whiteflies) suggests that this carrier retains an evolutionarily conserved housekeeping function (28, 30, 31, 50) and that bacteriocyte and symbiosomal membrane expression have been acquired to maximize the success of the endosymbiosis (70). ApNEAAT1 is a highly unusual transporter as, unlike the other characterized mammalian and arthropod AAAP carriers (28, 36, 39, 42, 63, 64), its function is not driven by the H^+^-electrochemical gradient (Fig. 4 and *SI Appendix*, Fig. S3). Rather, ApNEAAT1 transport is directed by local transmembrane amino acid concentration gradients. The acquisition of this particular AAAP transporter into the symbiosomal membrane thus likely provides both an evolutionary and an energetic advantage enabling bidirectional amino acid movement without energetic cost to local transmembrane ionic gradients.

Efficient utilization of their challenging food source, requires coordination of the aphid/*Buchera* genomes to produce complementary and integrated, rather than overlapping, biosynthetic pathways to produce vital components absent from diet (e.g., essential amino acids, vitamins) (20). Spatial separation of enzyme expression and activity within bacteriocyte compartments ensures that individual steps in metabolic pathways are partitioned between host and symbiont so that pathway completion is beneficial to both. Structural differences within the binding pockets of each transporter, a form of functional compartmentalization, produce distinct substrate selectivity (Fig. 4) (28), partitioning movement of different amino acids between diverse transport systems, reducing competition, and enabling selective provision of amino acids to discrete compartments to feed various biosynthetic and metabolic networks (20, 21, 28).

The absolute symbiotic interface, now known commonly as the symbiosomal or symbiosome membrane, was originally identified as the cytoplasmic or M3 membrane (as in the third membrane surrounding the symbiont) in the cabbage aphid *Brevicoryne brassicae* and pea aphid *A. pisum* (18, 73). The symbiosomal membrane is a common feature in insects, with up to 20% of all species considered to house endosymbiont-containing bacteriocytes (14). However, despite the key role played by this membrane in many endosymbioses, knowledge of how it enables transmembrane nutrient movement remains unknown. Here we report the localization and functional characteristics of the amino acid transporter ApNEAAT1. Ultimately, understanding the roles of ApNEAAT1, and the other transporters expressed in the symbiosomal membrane, in endosymbiosis, and the dynamic function of the symbiosomal membrane, are crucial for elucidating the cellular and molecular mechanisms that integrate hosts and endosymbionts, mechanisms that are foundational to the ecological and evolutionary success of many insect pests and vectors of human disease.

## Materials and Methods

### Materials.

[^3^H] and [^14^C] radiochemicals were from Hartmann Analytic, American Radiolabeled Chemicals, and PerkinElmer.

### Sequence and Threading Analyses.

PROMALS3D (54) was used (with default settings) for multialignment of full-length sequences. A sequence logo was created using WebLogo (55). Homology models of aphid ApNEAAT1 (ACYPI008971) and rat PAT2 (slc36a2) (both TM1 to TM10 only) against known APC superfamily crystal structures were constructed using the I-TASSER server (56) using default settings. The best fit “Model 1” for each of ACYPI008971 and PAT2 were aligned, using TM-Align from I-TASSER, with the highest scoring (TM score 0.908 and 0.863, respectively) structurally analogous crystal [*Escherichia coli* AdiC in an outward-open, arginine-bound conformation, PDB ID 3L1L (52) in both cases], to create figures in PyMOL (2.1.0 Open Source). Potential amino acid substrates were inserted into the binding pocket upon the arginine backbone in 3L1L using PyMOL. The positions of residues in TM3 were confirmed using HHPred (57) and Modeller (58) on the MPI Bioinformatics Toolkit (74).

### Preparation of Anti-ApNEAAT1 Antibody.

A monospecific anti-ApNEAAT1 antibody was produced as a custom antibody by Pacific Immunology Corp. A synthetic peptide corresponding to amino acids 356 to 370 of ApNEAAT1, plus a C-terminal cysteine (NTYMKKRVQNWDKTT-C), was synthesized and conjugated to maleimide-activated keyhole limpet hemocyanin (KLH). The KLH-coupled peptide was injected into New Zealand white rabbits for antibody production. Following a standard immunization protocol, monospecific anti-ApNEAAT1 antibodies were purified from rabbit serum using an affinity column with immobilized ApNEAAT1 peptide.

### Immunolocalizaton of ApNEAAT1 in Isolated Bacteriocyte Cells.

*A. pisum* clone LSR1 was maintained as a parthenogenetic lineage on *Vicia fabae* at 20 °C under a long-day photoperiod of 16 h of light to 8 h of darkness. Bacteriocytes were dissected from 10 to 15 young adult females in 0.9% (wt/vol) NaCl and fixed in 4% (wt/vol) formaldehyde (Thermo Scientific) overnight at 4 °C. Bacteriocytes were washed 5× (5 min per wash) in PBS at room temperature and then blocked with 5% (vol/vol) normal donkey serum (NDS; Jackson ImmunoResearch Laboratories) in PBS with 0.3% (vol/vol) Triton X-100 (PBST) for 1 h at room temperature. Samples were then incubated with primary anti-ApNEAAT1 antibody 1:500 in 5% NDS in PBST overnight at 4 °C. Bacteriocytes were washed 5× (5 min per wash), in PBS at room temperature and incubated with secondary Alexa-Fluor 568 donkey anti-rabbit IgG (H+L) antibody (Life Technologies) 1:1,000 in 5% NDS in PBST overnight at 4 °C. Bacteriocytes were washed 5× (5 min per wash) in PBS, and nuclei stained with DAPI (Life Technologies) at 300 nM for 30 min at room temperature. Bacteriocytes were mounted in 2,2′-thiodiethanol (Sigma-Aldrich) on a glass slide. Fluorescence images were acquired using a Leica TCS SP5 laser scanning confocal microscope. Control treatments were run in parallel and included localizations with peptide-preadsorbed primary antibody (using a 20-fold molar excess of peptide) and localizations with the secondary antibody only. The localization experiment with control treatments was repeated 3 times. In each experiment, multiple individual bacteriocytes were imaged in a single confocal plane.

### Functional Expression in *X. laevis* Oocytes.

The cloning of aphid transporter ApNEAAT1 (ACYPI008971) into plasmid pcDNA3.1 has been described previously (28). ApNEAAT1 was also amplified using Phusion High Fidelity DNA polymerase (Thermo Fisher) and directionally cloned into pCS2+ as a BamHI/Xho1 fragment. The use of PAT2 (rat slc36a2) in pSPORT has been described previously (39, 44). All constructs were sequenced fully. Plasmid DNA was linearized using HindIII (PAT2), NotI (pCS2+-ApNEAAT1), or BamH1 (pcDNA3.1-ApNEAAT1) and used as a template for cRNA synthesis. In vitro transcription was carried out using mMessage mMachine SP6 (pCS2+-ApNEAAT1), T7 (PAT2), or T7 Ultra (pcDNA3.1-ApNEAAT1) kits (Thermo Fisher). cRNA from either ApNEAAT1 construct gave equivalent levels of ApNEAAT1-functional expression in *X. laevis* oocytes. Female *X. laevis* were obtained from Xenopus1 and killed humanely in accordance with UK Home Office Schedule 1 directives. Alternatively, ovaries were purchased from the European *Xenopus* Resource Centre. Individual oocytes were recovered from ovarian tissue, as described previously (39, 63). Healthy stage V/VI oocytes were injected with 50 nL water or cRNA (0.5–1 µg/µL) using a Nanoinject II automated injector (Drummond Scientific Company). After injection, oocytes were maintained in Barth’s solution at 18 °C for 2 to 3 d before use in radiotracer uptake or electrophysiology experiments (39, 63).

### Transport Assays.

Amino acid uptake was measured, as described previously (39, 63). Negative control experiments were run in parallel, consisting of uptake into water-injected oocytes under identical conditions to those being tested with the cRNA-injected oocytes. Oocytes were washed in transport solution (39), then uptake of various [^3^H] or [^14^C] radiolabeled (1–5 µCi/mL) amino acids (10 µM unless stated otherwise) was measured at room temperature, over 20 to 40 min at pH 5.5, and in the absence of extracellular Na^+^ (choline chloride replacing NaCl in the transport solution) unless stated otherwise (see figure legends). These conditions give the greatest fold-uptake in other SLC36 AAAP transporters and here gave the greatest fold-uptake over water-injected (control) oocytes. Oocytes were then washed 3 times in ice-cold transport solution, lyzed in 10% SDS, and radioactivity quantified by scintillation counting. For efflux experiments (62), oocytes were preloaded with proline by microinjection of 50 nL [^3^H]proline (30 mM, 0.1 μCi/μL) resulting in [proline]_i_ ∼ 5 mM (assuming an effective oocyte volume of 250 nL). After a 10-min recovery period in modified Barth's solution (18 °C), oocytes were washed in transport solution and [^3^H]proline efflux measured (10 min) in the presence or absence of various extracellular amino acids (10 mM). The incubation solution was then removed for scintillation counting.

### Two-Electrode Voltage-Clamp Recordings.

Oocytes were placed in a Lucite chamber and perfused with Na^+^-free pH 5.5 uptake solution via a gravity-driven perfusion system. Chlorided silver wires served as recording electrodes. Intracellular microelectrodes (1–10 MΩ resistance) were pulled from borosilicate glass capillaries and filled with 1 M KCl. To allow direct comparison with uptake experiments, the membrane potential (V_M_) was clamped to resting V_M_, which in Na^+^-free, pH 5.5 conditions was −30 mV, with a 2-electrode voltage clamp amplifier (Warner Instruments). Transmembrane currents (I_M_) were low-pass filtered at 1 kHz (LPF-202, Warner Instruments) and recorded by a strip-chart recorder (Kipp & Zonen). Current traces were digitized using Inkscape (v0.91). All recordings were performed at room temperature. The current induced by various amino acids was calculated as the difference between I_M_ before amino acid exposure (baseline) and I_M_ 60 s into amino acid exposure.

### Data and Statistical Analysis.

Transport data are mean ± SEM and are typically expressed as pmol.oocyte^−1^.[duration]^−1^. For transporter-specific uptake, uptake into water-injected oocytes (measured under identical conditions) was subtracted from the total uptake. Curve fitting (Michaelis–Menten kinetics), statistical analysis and graph preparation were carried out using GraphPad Prism 6. Two-way ANOVA was used to compare mean values with Tukey’s or Sidak’s multiple comparisons posttests, unless stated otherwise. Statistics are described in the figure legends.

## Supplementary Material

Supplementary File
