# Supplementary material for: Trading amino acids at the aphid–Buchnera symbiotic interface
Source: Proc Natl Acad Sci U S A. 2019 Jul 23;116(32):16003–11. doi: 10.1073/pnas.1906223116 (PMC6690024; doi:10.1073/pnas.1906223116)
Supplement: Supplementary File [file pnas.1906223116.sapp.pdf]

PNAS
www.pnas.org

Supplementary Information for

Trading amino acids at the aphid/*Buchnera* symbiotic interface

Honglin Feng, Noel Edwards, Catriona M. H. Anderson, Mike Althaus, Rebecca P. Duncan, Yu-Ching Hsu, Charles W. Luetje, Daniel R. G. Price, Alex C. C. Wilson and David T. Thwaites

Corresponding authors
Alex Wilson: acwilson@miami.edu
David T. Thwaites: d.t.thwaites@ncl.ac.uk

This PDF file includes:

Figs. S1 to S4
Table S1
References for SI reference citations

www.pnas.org/cgi/doi/10.1073/pnas.1906223116

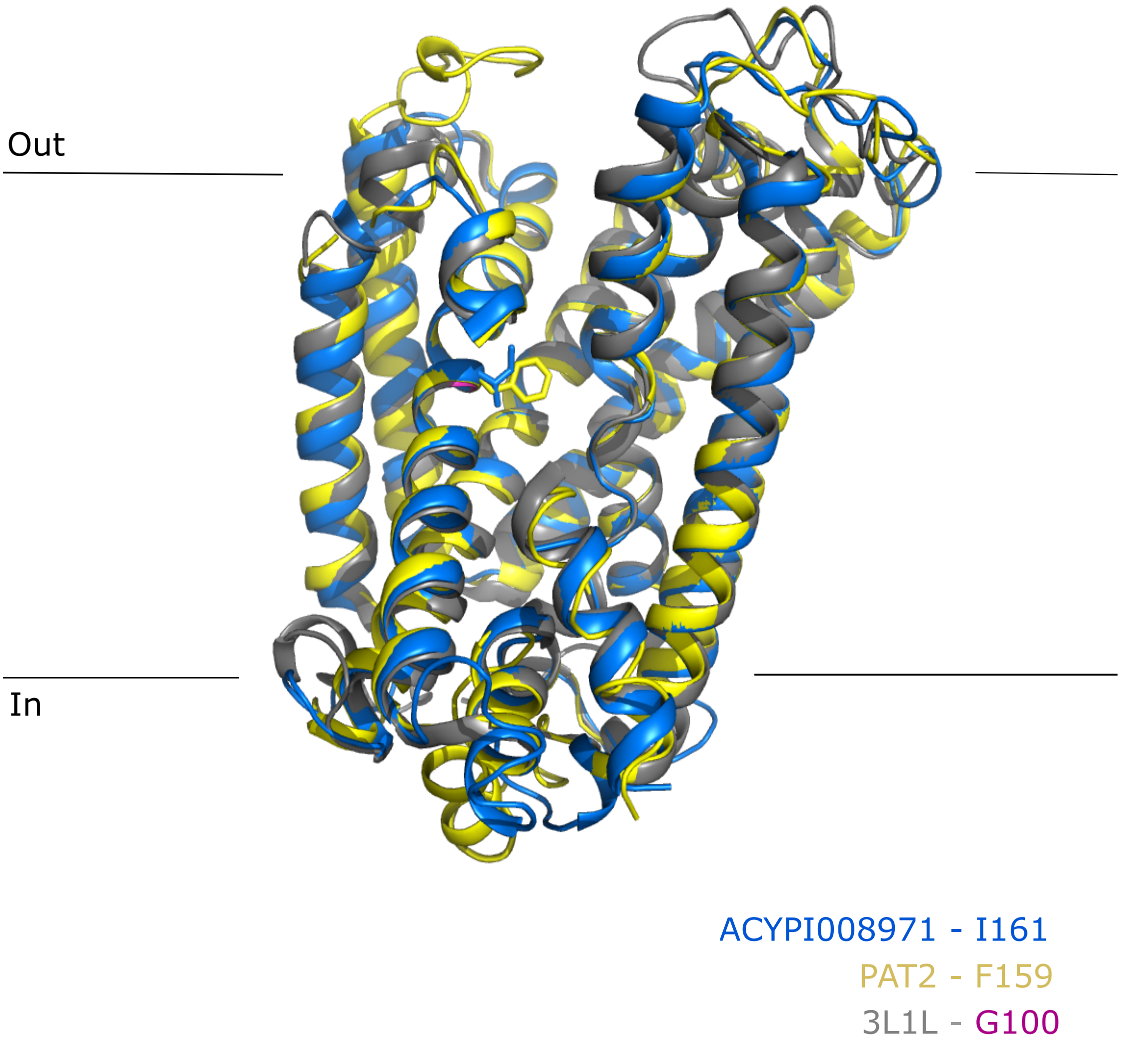

Fig. S1. I161 in ACYPI008971 (ApNEAAT1) occupies the same position as F159 in PAT2. Homology models of ACYPI008971 (blue) and PAT2 (slc36a2, yellow) produced from the core 5+5 TM motif (TM1-10) of each protein using the I-TASSER server. Models are shown superimposed upon TM1-10 of the top-scoring crystal, 3L1L (grey), which is of the amino acid transporter AdiC from *E.coli* (1). The orientation is side-on. The residues of interest, I161 (ACYPI008971) and F159 (PAT2) of TM3, are shown as sticks projecting into the central part (binding pocket) of the proteins. A small section of TM10 has been omitted from each model and the crystal to allow a clearer view of these residues. ACYPI008971 I161 and PAT2 F159 occupy the same position as G100 in AdiC (magenta segment of TM3 ribbon). We have previously shown that F159 and G100 occur in the same position as each other and that this is equivalent to V104 in LeuT (2). V104 forms part of the binding pocket in LeuT (2, 3).

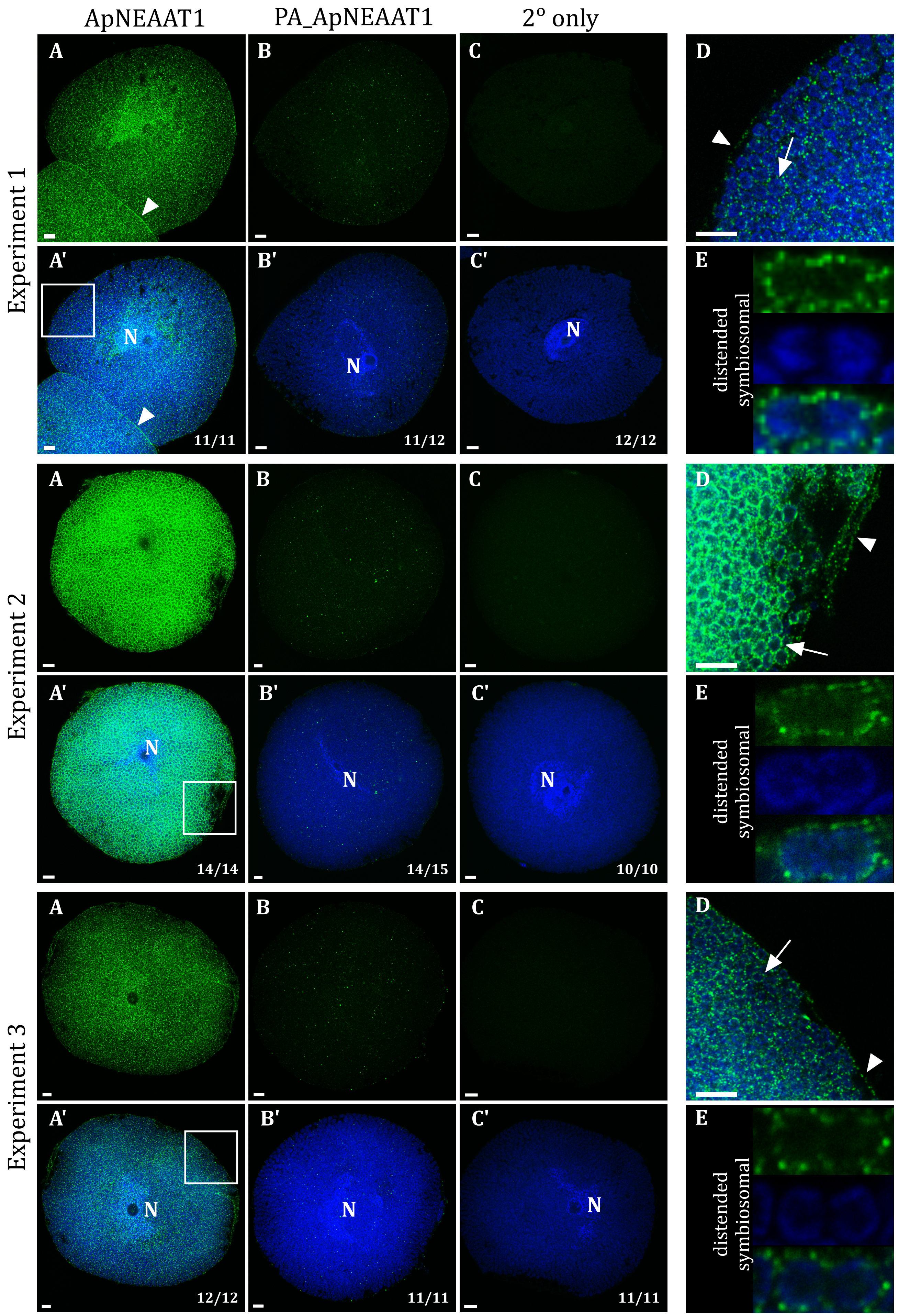
ApNEAAT1
PA_ApNEAAT1
2° only
Experiment 1
Experiment 2
Experiment 3
A
B
C
D
A'
B'
C'
E
N
distended symbiosomal
11/11
11/12
12/12
14/14
14/15
10/10
12/12
11/11
11/11

Fig. S2. Quantification of ApNEAAT1 localization in isolated bacteriocyte cells from three independent experiments. Within each experiment: (*A*) localization pattern of anti-ApNEAAT1 antibody (green) in isolated bacteriocyte cells; (*A′*) merge of the anti-ApNEAAT1 image and DAPI-stained DNA (blue); (*B*–*B′*) comparable control experiments were performed with isolated bacteriocytes with peptide pre-adsorbed (PA) anti-ApNEAAT1antibody. (*C*-*C′*) Control experiments where performed with isolated bacteriocytes with secondary antibody only (2° only). (*D*) Magnified region of bacteriocyte cell showing merge of anti-ApNEAAT1 localization (green) and DAPI-stained DNA (blue), arrow head marks localization to the bacteriocyte cell membrane, arrow marks localization to the symbiosomal membrane. (*E*) Localization to the distended symbiosomal membrane was observed in 1/11 bacteriocytes in Experiment 1, 9/14 bacteriocytes in Experiment 2, and 2/12 bacteriocytes in Experiment 3. The secondary antibody is Alexa-Fluor 568 donkey anti-rabbit IgG (H+L). For all images, a single representative confocal plane is shown and the quantification data are shown in the lower right corner of each panel representing the number of cells showing similar staining pattern out of the number of cells assayed in the same experiment. Scale bars = 10 μm. N, bacteriocyte cell nucleus. For each experiment, boxes in *A* indicate location of *D*.

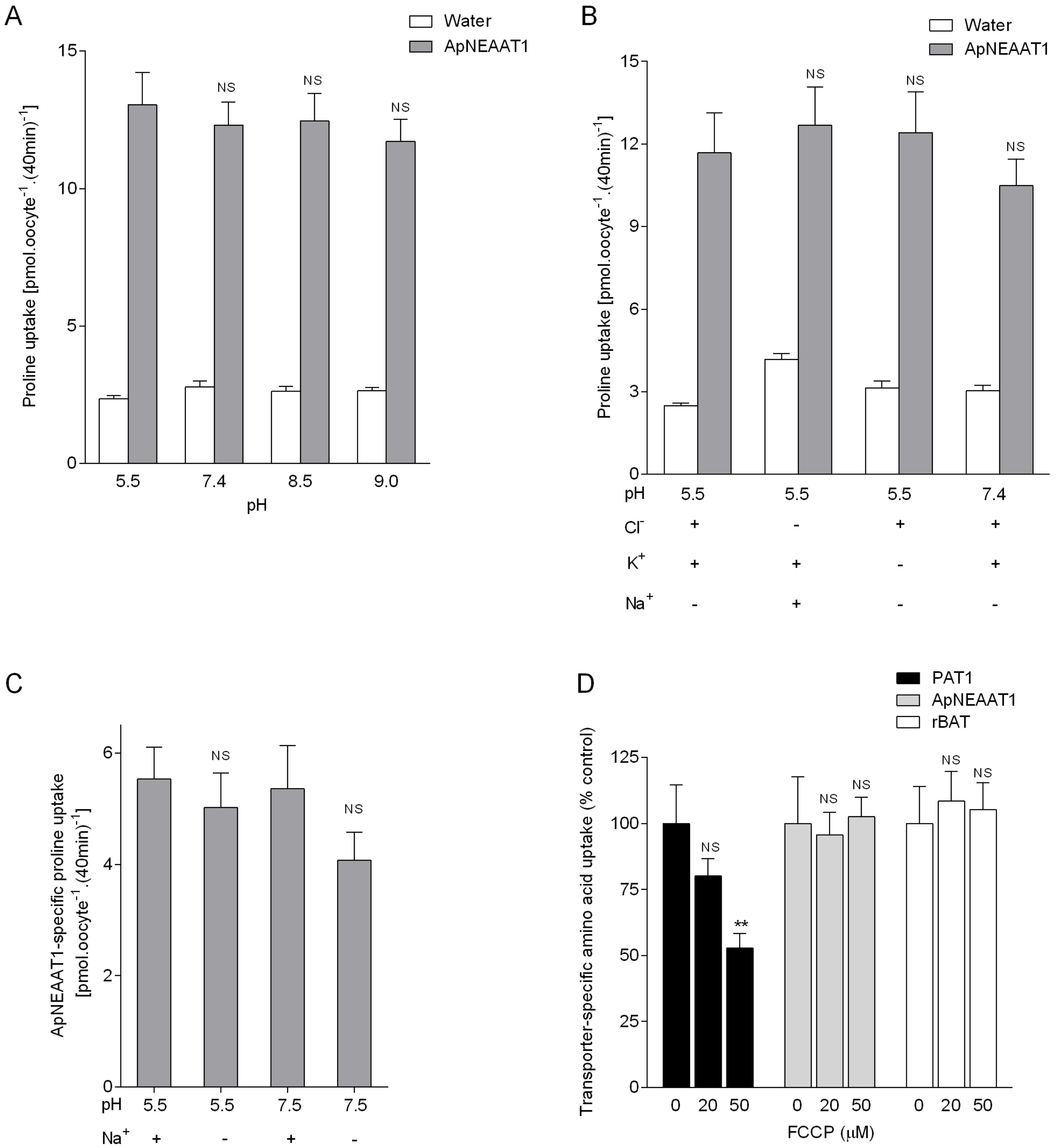

A
Water
ApNEAAT1
Proline uptake [pmol.oocyte-1.(40min)-1]
0
5
10
15
NS
NS
NS
5.5
7.4
8.5
9.0
pH
B
Water
ApNEAAT1
Proline uptake [pmol.oocyte-1.(40min)-1]
0
3
6
9
12
15
NS
NS
NS
pH 5.5 5.5 5.5 7.4
Cl- + - + +
K+ + + - +
Na+ - + - -
C
ApNEAAT1-specific proline uptake [pmol.oocyte-1.(40min)-1]
0
2
4
6
NS
NS
pH 5.5 5.5 7.5 7.5
Na+ + - + -
D
PAT1
ApNEAAT1
rBAT
Transporter-specific amino acid uptake (% control)
0
25
50
75
100
125
NS
**
NS
NS
NS
NS
0 20 50 0 20 50 0 20 50
FCCP (μM)

Fig. S3. Amino acid uptake via ApNEAAT1 is insensitive to extracellular pH, Na^+, K^+ and Cl^- and to the disruption of the H^+-electrochemical gradient. Amino acid uptake was measured over 20-40 min as described in the main text. (*A*) Proline uptake measured in Na^+-free conditions, pH 5.5-9.0, in ApNEAAT1-expressing and water-injected (control) oocytes. For measurements at pH 8.5 and pH 9.0, HEPES was replaced by Tris base. n = 10. NS, $P > 0.05$ vs. pH 5.5 (two-way ANOVA with Tukey's post-test). (*B*) Proline uptake measured in the presence (+) and absence (-) of extracellular K^+, Na^+ and/or Cl^-. For Cl^--free solutions, D-gluconic acid replaced chloride. For K^+-free solutions, KCl was omitted. n = 10. NS, $P > 0.05$ vs. Na^+-free, pH 5.5 control (two-way ANOVA with Tukey's post-test). (*C*) Proline uptake measured in the presence (+) and absence (-) of extracellular Na^+ at both pH 5.5 and pH 7.5. Uptake into water-injected oocytes was subtracted to give ApNEAAT1-specific uptake. n = 20. NS, $P > 0.05$ vs. $+Na^+$ (one-way ANOVA with Tukey's post-test). (*D*) Proline (10 µM) uptake into oocytes expressing ApNEAAT1 or PAT1 (mouse slc36a1), and arginine (10 µM) uptake into oocytes expressing the heavy-chain rBAT (human SLC3A1, which leads to surface expression of an endogenous, H^+-independent, *Xenopus* amino acid transporter) was measured in Na^+ free, pH 5.5 uptake conditions. Oocytes were incubated in the presence (20 or 50 µM) or absence (0 µM) of the proton ionophore FCCP (carbonyl cyanide 4-(trifluoromethoxy)phenylhydrazone) for 20 min prior to, and throughout, uptake. Uptake into water-injected oocytes was subtracted to give transporter-specific uptake. n = 9-10. **, $P < 0.01$; NS, $P > 0.05$ vs. absence of FCCP (two-way ANOVA with Tukey's post-test). The use of PAT1 in pCRII-TOPO has been described previously (4, 5). rBAT (human SLC3A1) in pSPORT was a gift from H. Murer (University of Zürich, Switzerland). *In vitro* transcription of PAT1 and rBAT was carried out as for PAT2, as described in the main text.

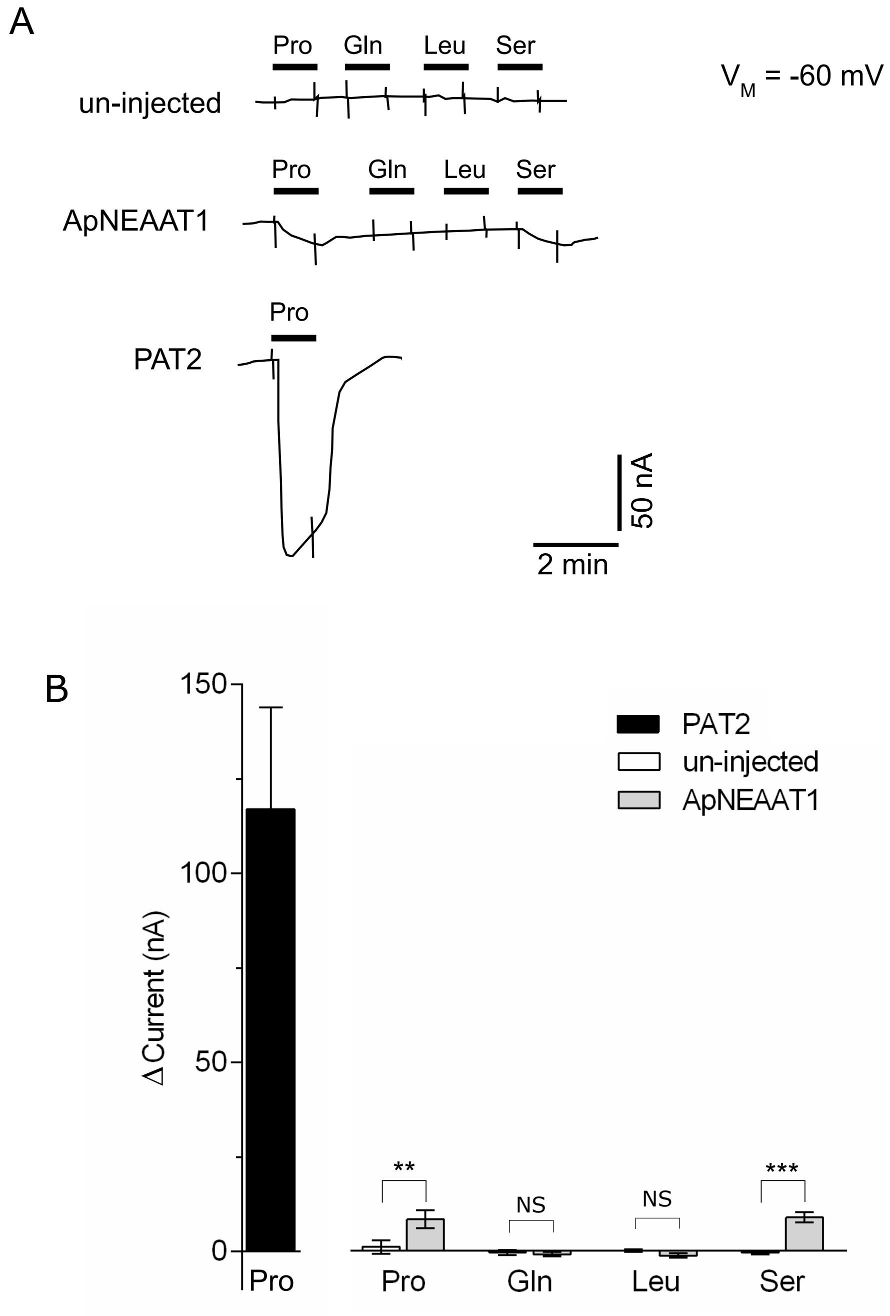

Fig S4. ApNEAAT1 displays small currents at hyperpolarised membrane potentials reminiscent of non-stoichiometric, inward cationic currents found in certain electroneutal mammalian AAAP transporters (6-8). TEVC was used to measure currents associated with different amino acids (10 mM) in ApNEAAT1 -expressing, PAT2 -expressing and un-injected (control) oocytes clamped at a V_M of -60 mV in Na^+-free pH 5.5 conditions. (*A*) Example traces showing small, slow and poorly-reversing currents elicited by a saturating concentration (10 mM) of substrates (Pro, Ser) but not non-substrates (Gln, Leu) of ApNEAAT1. Transport-associated conductances have been observed in electroneutral transporters that do not carry out net charge movement (6-8). Under the same conditions as for ApNEAAT1, Pro induces large currents in PAT2 -expressing oocytes, similar to those measured at resting membrane potential (-30 mV, Fig 4*F* and Table S1) in keeping with the 1:1 stoichiometry of PAT2 (4). (*B*) Mean data show small, yet significant, substrate-induced currents in ApNEAAT1 oocytes. The current associated with various amino acids was calculated as the difference between I_M before amino acid exposure (baseline) and I_M 60 s into amino acid exposure. n = 4. NS, $P > 0.05$; **, $P < 0.01$; ***, $P < 0.001$; ApNEAAT1 vs. un-injected (two-way ANOVA with Tukey's post-test comparing only ApNEAAT1 vs. un-injected).

Table S1. Unlike the H^+-coupled amino acid transporter PAT2, ApNEAAT1-mediated transport is not associated with inward currents when oocytes were clamped at resting membrane potential. Proline-associated inward current was assessed by TEVC in oocytes clamped at resting V_M (-30 mV), superfused with Na^+-free, pH 5.5 solution and exposed to proline (0.1-1 mM). Example traces can be seen in Fig. 4*F*. n = 5. NS, $P > 0.05$; *, $P < 0.05$; **, $P < 0.01$; ***, $P < 0.001$ vs. un-injected (two-way ANOVA with Tukey's post-test).

	Δ Current (nA)		
[Proline] (mM)	Un-injected	ApNEAAT1	PAT2
0.1	-0.2 ± 1.4	0.2 ± 1.8 NS	66 ± 19 *
0.3	3.8 ± 0.9	2.0 ± 0.8 NS	93 ± 29 **
1	1.2 ± 0.6	2.8 ± 1.3 NS	104 ± 33 ***

References for Supplemental Information

1. Gao X, *et al.* (2010) Mechanism of substrate recognition and transport by an amino acid antiporter. Nature 463:828-832.

2. Yamashita A, Singh SK, Kawate T, Jin Y, Gouaux E (2005) Crystal structure of a bacterial homologue of Na^+/Cl^--dependent neurotransmitter transporters. Nature 437:215-223.

3. Edwards N, *et al.* (2018) Resculpting the binding pocket of APC superfamily LeuT-fold amino acid transporters. Cell Mol Life Sci 75:921-938.

4. Boll M, Foltz M, Rubio-Aliaga I, Kottra G, Daniel H (2002) Functional characterization of two novel mammalian electrogenic proton-dependent amino acid cotransporters. J Biol Chem 277:22966-22973.

5. Kennedy DJ, Gatfield KM, Winpenny JP, Ganapathy V, Thwaites DT (2005) Substrate specificity and functional characterisation of the H^+/amino acid transporter rat PAT2 (Slc36a2). Br J Pharmacol 144:28-41.

6. Bröer A, *et al.* (2002) Regulation of the glutamine transporter SN1 by extracellular pH and intracellular sodium ions. J Physiol 539:3-14.

7. Chaudhry FA, Reimer RJ, Edwards RH (2002) The glutamine commute: take the N line and transfer to the A. J Cell Biol 157:349-355.

8. Schneider HP, Bröer S, Bröer A, Deitmer JW (2007) Heterologous expression of the glutamine transporter SNAT3 in *Xenopus* oocytes is associated with four modes of uncoupled transport. J Biol Chem 282:3788-3798.
